# Ascorbic Acid Sensitizes Colorectal Carcinoma to the Cytotoxicity of Arsenic Trioxide *via* Promoting Reactive Oxygen Species-Dependent Apoptosis and Pyroptosis

**DOI:** 10.3389/fphar.2020.00123

**Published:** 2020-02-21

**Authors:** Wei Tian, Zhuo Wang, Nan-nan Tang, Jia-tong Li, Yu Liu, Wen-Feng Chu, Bao-Feng Yang

**Affiliations:** Department of Pharmacology, The State-Province Key Laboratories of Biomedicine Pharmaceutics of China, Key Laboratory of Cardiovascular Research, Ministry of Education, College of Pharmacy, Harbin Medical University at Harbin, Heilongjiang, China

**Keywords:** colorectal cancer, ascorbic acid, arsenic trioxide, apoptosis, pyroptosis, reactive oxygen species

## Abstract

Arsenic trioxide (ATO) is an effective therapeutic agent against acute promyelocytic leukemia (APL); however, its anti-tumor effect on solid tumors such as colorectal cancer (CRC) is still in debate. Ascorbic acid (AA) also produces a selective cytotoxic activity against tumor cells. Here, we exploit the potential benefit of ATO/AA combination in generating cytotoxicity to CRC cells, which may lay the groundwork for the potential combinational chemotherapy of CRCs. According to the results, we found that ATO and AA effectively inhibited the viability of human CRC cells in a synergistic manner. AA and ATO corporately activated caspase-3 to trigger apoptosis and upregulated the expression of caspase-1 and promoted formation of inflammasomes to induce pyroptosis. Furthermore, the stimulation of reactive oxygen species (ROS) overproduction was demonstrated as a subcellular mechanism for apoptosis and pyroptosis induced by ATO/AA combination treatment. Our findings suggest that ATO combination with a conventional dosage of AA offers an advantage for killing CRC cells. The synergistic action of ATO/AA combination might be considered a plausible strategy for the treatment of CRC and perhaps other solid tumors as well.

## Introduction

Colorectal cancer (CRC) is the third most commonly diagnosed malignancy and the fourth leading cause of cancer-related deaths in the world (Arnold et al., 2017). It is the third most frequent cancer in men after lung and prostate cancer, and is the third most frequent cancer in women after lung and breast cancer (Siegel et al., 2018). Existing treatment options include surgery, radiation therapy, and chemotherapy, each with trade-offs between disease treatment and quality of life due to various side effects (Zerillo et al., 2017). In particular, resistance to chemotherapy is intimately related to poor clinical outcomes. Arsenic compounds have been used for more than 2,400 years in treating a variety of ailments in both western and traditional Chinese medicines (Hughes, 2002). In the 1970s, Zhang and colleagues first demonstrated the effectiveness of arsenic in curing acute promyelocytic leukemia (APL), a M3 subtype of acute myeloid leukemia (AML). Subsequent basic and clinical studies confirmed the efficacy of arsenic trioxide (ATO) in the treatment of APL. It has been reported that APL patients can achieve a complete alleviation with ATO with minimal side effects (Mathews et al., 2010). Later on, ATO was found to induce apoptosis in several solid tumors such as in human lung, breast cancer cells, presumably *via* inducing overproduction of reactive oxygen species (ROS) (Waxman and Anderson, 2001; Pettersson et al., 2009; Martin-Martin et al., 2016); yet treatment with ATO alone did not benefit patients clinically (Subbarayan and Ardalan, 2014). Moreover, the required doses of ATO increased the risk of side effects such as its cardiotoxicity, including long QT syndrome or torsade de pointes leading to sudden cardiac death (Chu et al., 2012). Nonetheless, when combined with other agents, ATO can produce therapeutic benefits with governable toxicity. It is believed that high dose requirement and drug resistance cause failure of ATO treatment of solid tumors. Clearly, new strategies are needed to enhance the antitumor activity of ATO and/or to eliminate drug resistance while reducing the required dose of ATO to minimize its side effects.

Ascorbic acid (AA), also known as vitamin C, is often used as an antioxidant at low concentrations. Numerous studies, however, have uncovered its pro-oxidant activity at higher concentrations (De Laurenzi et al., 1995; Schwartz, 1996; Mandl et al., 2009; Vuyyuri et al., 2013). Prominently, AA has found its therapeutic potential for cancers (McConnell and Herst, 2014). A number of clinical trials have generated the data ascertaining the therapeutic value of AA in patients with terminal cancers (Cameron and Pauling, 1976; Moertel et al., 1985; Hoffer et al., 2008; Stephenson et al., 2013). It has been proposed that AA can produce a selective cytotoxic activity against tumor cells without affecting normal cells (Xia et al., 2017). Moreover, overwhelming reports have demonstrated that combinations of pharmacological AA with other anticancer agents can enhance cytotoxicity (Ma et al., 2014).

Apoptosis (programmed cell death) is among the most common mechanisms for counteracting cancer cells or sensitizing cancer cells to chemotherapeutic agents and radiation therapy (Ghobrial et al., 2005). ROS is known to be a strong apoptotic inducer involved in a variety of pathological processes (Chen et al., 2017). The oxidative stress caused by ROS thus can be utilized as a novel cancer-damaging adjuvant (Ghobrial et al., 2005). Many studies have indicated that agents interfering ROS metabolism can selectively eradicate the cancer cells by elevating the accumulation of ROS above a threshold of toxicity (Li et al., 2016).

In addition to apoptosis, pyroptosis as another form of programmed cell death, is also believed to be one of the mechanisms for cancer cell death (Derangere et al., 2014). Pyroptosis is lytic cell death initiated by inflammasomes, a process involving the activation of caspase-1 or caspase-11/4/5 to cleave gasdermin D (GSDMD) (He et al., 2015). It has been proposed that pyroptosis can be utilized for cancer therapy (Wang et al., 2019). Intriguingly, ROS can evoke the inflammasome-dependent pyroptosis in addition to induce apoptosis (Chen and Nunez, 2011; Lin and Zhang, 2017).

We therefore set up the present study to exploit the feasibility of combination of ATO and AA for better anti-cancer efficacy and delineate the possible roles of apoptosis and pyroptosis in conferring the advantages to the combination over each of the single agents. Additionally, two kinds of CRC cells were employed to explore the anti-tumor efficacy of the combination of AA and ATO. Specifically, we investigated the potential facilitating effects of AA on the anti-tumor property of ATO in solid tumor cells or CRC cells or the synergistic effects of ATO/AA combination, and the underlying cellular mechanisms for the effects.

## Materials and Methods

### Cell Culture and Reagents

Human SW620 colorectal adenocarcinoma (ATCC^®^ CCL-227™) and LOVO colorectal adenocarcinoma (ATCC^®^ CCL-229™) cell lines were purchased from the American Type Culture Collection (ATCC, Manassas, VA, USA). LOVO cells were maintained in F-12K medium (SH30526.01; Hyclone, Utah, USA), supplemented with 10% fetal bovine serum (FBS) and 1% antibiotic–antimycotic solution (Beyotime, Shanghai, China) at 37°C in 5% CO_2_. SW620 cells were cultured in L-15 medium (SH30525.01; Hyclone) containing 2 mM glutamine, 10% FBS, and 1% antibiotic–antimycotic solution with a humidified atmosphere containing 100% air at 37°C. FBS was purchased from BI (04-001-1A; BioInd, Kibbutz, Israel).

Cells were treated with varying concentrations of AA in combination with a concentration (2 μM) of ATO for 24 h to induce apoptosis. Only sub-confluent monolayers of cells (70% to 80%) were used in all experiments. Arsenious acid and sodium chloride injection was obtained from Harbin Medical University Pharmaceutical Co., Ltd (Harbin, China). AA was purchased from Sigma-Aldrich (A5960; USA) and dissolved in phosphate-buffered saline (PBS) buffer solution (SH30256.01B; Hyclone, USA). Tempol (2226-96-2; USA) and Z-VAD-FMK (187389-52-2; USA) were obtained from MedChemexpress (Monmouth Junction, NJ, USA). The antibodies against Bax (#5023), Bcl-2 (#2872), caspase-3 (#9662), cleaved-caspase-3 (#9662), and caspase-1 (#3866) were purchased from Cell Signaling Technology (Boston, Massachusetts, USA).

### Cell Viability Assay

Cell viability was evaluated using the Cell Counting Kit-8 (CCK-8, LJ621; Dojindo, Japan) according to the manufacturer’s instructions. Cells were seeded in 96-well flat bottom microtiter plates at a density of 5,000 cells per well. Cells were starved for 8 h and then treated with AA and ATO for 24 h. After drug treatment, cells were incubated with 10 μl CCK-8 solution for 1.5 h. The absorbance was measured at 450 nm on a microplate spectrophotometer (Tecan, Mannedorf, Switzerland).

### Combination Index Analysis

The combination index (CI) of growth inhibition was calculated using the CompuSyn software (Biosoft, USA). The CI was calculated to determine the synergistic cytotoxicity according to the classic isobologram equation: CI = (D)1/(Dx)1 + (D)2/(Dx)2, where Dx is the concentration of one drug that produces the effect, and (D)1 and (D)2 are the concentrations of the two drugs in combination that produce the same effect. CI = 1 indicates an additive effect, CI < 1 indicates synergy, and CI > 1 reflects antagonism between two drugs. Combination studies were performed using the similar protocol as described in the previous study (Tan et al., 2019). Briefly, the IC50 of ATO and AA was first determined in single drug experiments. Cells were then treated with increasing doses of ATO or AA at a non-constant-ratio combination of both drugs. The cell growth of single and combination drug experiments were determined after 24 h.

### TdT-Mediated dUTP Nick-End Labeling Assay

TdT-mediated dUTP nick-end labeling (TUNEL) assay was performed in SW620 cells plated in 24-well chamber slides treated with either 2 mM AA or 2 μM ATO or combination of AA and ATO for 24 h. Cells were fixed in 4% methanol-free formaldehyde solution in PBS (25 min at 4°C), washed twice with PBS, permeabilized with 0.2% Triton X-100 (5 min), and rinsed twice with PBS. The SW620 cells were analyzed for apoptosis with TUNEL assay by using *In Situ* Cell Death Detection Kit (Cat. No. 11684817910; Roche, Mannheim, Germany).The cells were incubated with TUNEL Reaction Mixture for 1 h at 37°C in the dark. After counterstaining with DAPI at room temperature for 10 min, analysis was performed using a fluorescence microscope (Zeiss, Jena, Germany). TUNEL-positive cells were stained in red within the nucleus. The assay was repeated five times.

### Electron Microscopy

Cells were harvested and fixed in 2.5% glutaraldehyde (pH 7.4) overnight and then immersed in 0.1 M cacodylate buffer with 1% osmium tetroxide for 1 h. Samples were dehydrated with a concentration gradient of ethanol and then embedded in Epon medium and dissected into 60–70 nm sections. After being stained with uranylacetate and lead citrate, sections were examined under a JEOL 1200 electron microscope (JEOL Ltd., Tokyo, Japan).

### Apoptosis Assay

The Annexin V–FITC apoptosis detection kit purchased from Beyotime Biotechnology was applied to detect cell apoptosis. SW620 cells (5.0 × 10^5^/1 ml) in logarithmic phase were treated with different drugs for 24 h. The treated cells were collected and washed with cold PBS after treatment. In accordance with the manufacturer’s instructions, the cells were stained with Annexin V–FITC and propidium iodide for 20 min at room temperature in the dark, and the apoptosis rate of the treated cells was determined immediately after staining by CytoFlex Flow cytometry (CytoFLEXS; Beckman Coulter, United States).

### Acridine Orange/Ethidium Bromide Staining Assay

Acridine orange/ethidium bromide (AO/EB) apoptotic staining was used to detect the morphology of apoptotic cells. SW620 cells were plated into 24-well chamber slides treated with either AA or ATO or combination of AA and ATO for 24 h. Then the cells were washed three times in PBS at room temperature, and then mixed with 1 ml of dye mixture containing 100 μg/ml AO and 100 μg/ml EB (CA1140; Solarbio, China) in PBS for 5 min. Cellular morphological changes was visualized immediately using a fluorescence microscope (Zeiss, Jena, Germany). Viable cells stained only by AO appeared bright green with intact structure, whereas apoptotic cells were stained orange with fragmented chromatin. And necrotic cells were stained red with condensation of chromatin by EB.

### Clonogenic Assay

The clonogenic assay was used for accessing the proliferation of SW620 cells after treating with ATO, AA, and ATO/AA combination. For clonogenic assay, after 24 h of ATO, AA, and ATO/AA combination treatment, single cell suspension of 1,000 cells were plated in 20-mm dishes. Then the dishes were incubated in a 5% CO2 incubator at 37°C for 15 days. At day 15 after the colonies were formed, each dishes was washed in PBS and fixed with 4% paraformaldehyde at room temperature for 5 min. Then, the colonies were stained with 0.5% crystal violet for 30 min, washed, dried, and imaged. The number of colonies containing more than 50 cells were counted and calculated under a light microscope and used as an index for clonogenicity. The quantification of colony was determined by using Image J software. Each experiment was performed in triplicate.

### Determination of ROS Generation

Intracellular ROS was detected by means of an oxidation-sensitive dihydroethidium (DHE) fluorescent probe (Beyotime Biotechnology) using fluorescence microscope. After treatment with ATO (2 μM) and/or AA (2 mM), with or without Tempol (1 mM) for 24 h, cells were washed twice in PBS. Following treatment, floating and adherent cells were harvested, and they were then incubated with 10 μM DHE at 37°C for 20 min according to the manufacturer’s instructions. Then analysis was performed using a fluorescence microscope (Zeiss, Jena, Germany) to detect intracellular ROS levels. We also used flow cytometry to detect intracellular ROS levels. After treatment, floating, and harvesting cells, 10 μM DCFH-DA (Beyotime Biotechnology) was added to the cells and the cells were further incubated for 60 min. DCFH-DA was deacetylated intracellularly by nonspecific esterase, which was further oxidized by ROS to the fluorescent compound 2,7-dichlorofluorescein (DCF). DCF fluorescence distribution of 20,000 cells was detected by CytoFlex Flow cytometry (CytoFLEXS; Beckman Coulter, United States) at an excitation wavelength of 488 nm and an emission wavelength of 535 nm.

### Western Blot Assay

Cells were harvested from cultured dishes and lysed in a RIPA lysis buffer (Beyotime, China) with protease (Roche, Germany). Protein concentration was determined using a BCA Protein Assay Kit (P0009; Beyotime). Cell lysates (100 μg protein/lane) were separated on a 12% Tris-Tricine SDS-PAGE gel for nitrocellulose membrane (PALL, Germany) blotting. The blotted membranes were blocked with 5% skim milk for 1 h and then incubated with primary antibodies at 4°C overnight. After incubation with secondary antibody with gentle shaking at room temperature for 30–60 min, the membranes were scanned by Odyssey Infrared Imaging System (Li-COR, USA). Band density was measured by densitometry, quantified using gel plotting macros of NIH image 1.62, and normalized to β-actin as an internal control.

### Quantitative Real-Time PCR

Total RNA was extracted from pretreated cells with TRIzol reagent (Invitrogen Carlsbad, CA, USA). The cDNAs were produced from the RNA samples with Reverse Transcription Kit (Toyobo, Shanghai, China) and RNA specific Bulge-loop™ RNA RT primers (Invitrogen Carlsbad, CA, USA). GAPDH mRNA was used as an endogenous control for data normalization. Real-time PCR was performed on the ABI Prism 7500 Sequence Detection System (Applied Biosystems) using SYBR Green I Real-Time PCR kit (Roche Diagnostics, Mannheim, Germany). Relative mRNA expression was determined using the Ct method. All experiments were performed at least five times. The following primers sequence:

Interleukin (IL)-18: forward 5′-CTTCCAGATCGCTTCCTCTC-3′ and reverse 5′-TCAAATAGAGGCCGATTTCC-3′.
IL-1β: forward 5′-TTTGAGTCTGCCCAGTTCCC-3′ and reverse 5′-TCAGTTATATCCTGGCCGCC-3′.
GAPDH: forward 5′-GAAGGTGAAGGTCGGAGTCA-3′ and reverse 5′-AATGAAGGGGTCATTGATGG-3′.

### Statistical Analysis

Data are presented as mean ± standard error ofthe mean (SEM) for three independent experiments. Comparisons of mean values were performed with one-way ANOVA followed by the Tukey procedure. *P* < 0.05 was considered statistically significant. Statistical analysis was performed using Prism 7.0c GraphPad Software (GraphPad, San Diego, CA, United States).

## Results

### AA Enhances the Cytotoxicity of ATO in CRC Cells

**Figure 1:**
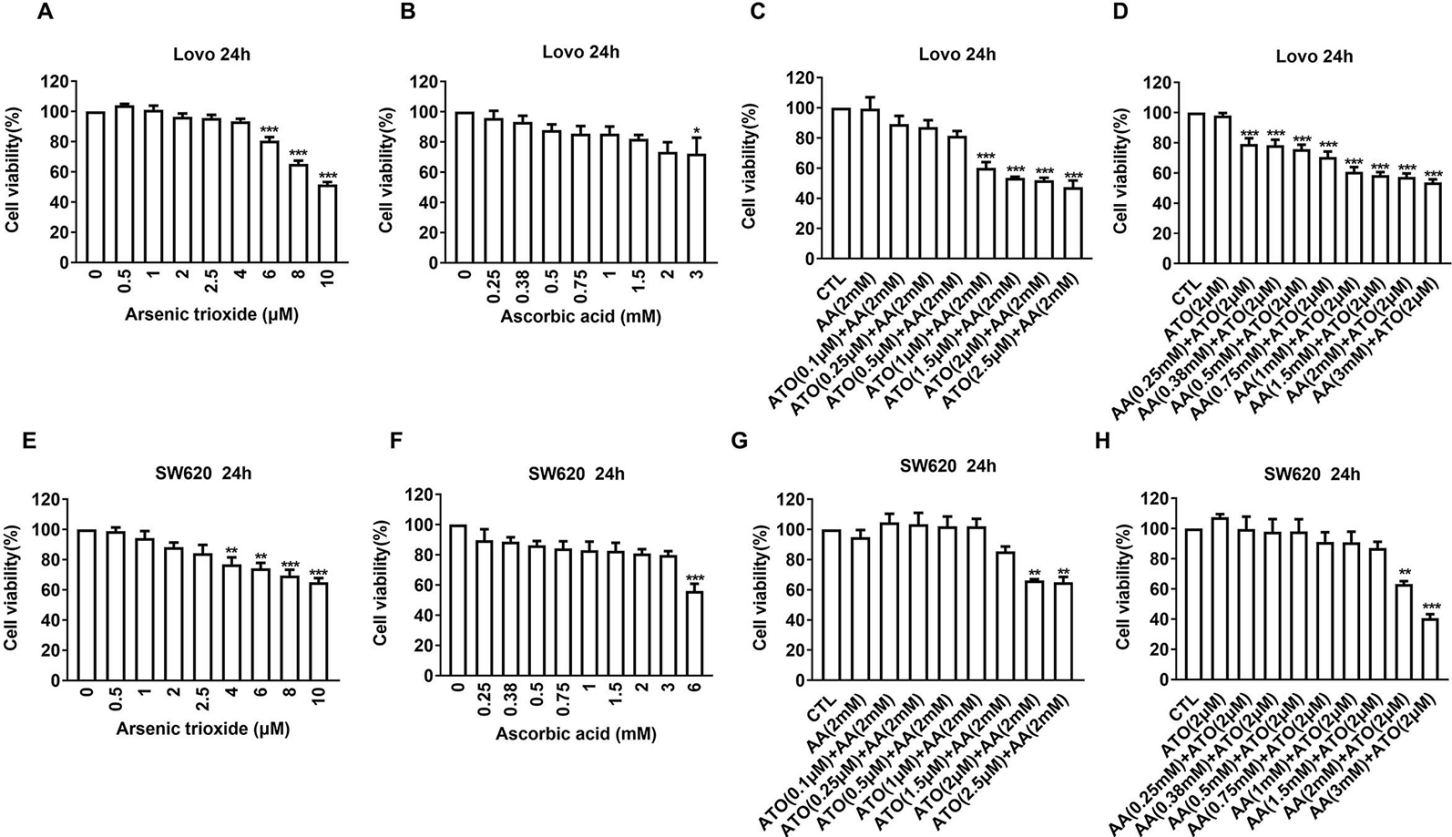
Effects of ascorbic acid (AA), arsenic trioxide (ATO) alone, or co-administration on viability of colorectal cancer (CRC) cell lines. **(A)** Inhibitory effects of ATO alone on viability of LOVO cells. **(B)** Inhibitory effects of AA alone on viability of LOVO cells. **(C)** Facilitating effects of ATO on the suppression of LOVO cell viability induced by AA (2 mM). **(D)** Facilitating effects of AA on the suppression of LOVO cell viability induced by ATO (2 μM). **(E)** Inhibitory effects of ATO alone on viability of SW620 cells. **(F)** Inhibitory effects of AA alone on viability of SW620 cells. **(G)** Facilitating effects of ATO on the suppression of SW620 cell viability induced by AA (2 mM). **(H)** Facilitating effects of AA on the suppression of SW620 cell viability induced by ATO (2 μM). Relative or percent cell viability was determined by Cell Counting Kit-8 (CCK-8) assay. Data are expressed as mean ± SEM of three independent experiments. *P < 0.05, **P < 0.01, ***P < 0.001 by one-way ANOVA.

First, we evaluated the effects of ATO or AA alone on the viability of CRC cell lines using CCK-8 assay. We observed that ATO at concentrations ≥6 μM and AA ≥3 mM significantly decreased the number of living LOVO cells (**Figures 1A, B**). Addition of ATO to culture medium in the presence of 2 mM AA facilitated the inhibitory effect of AA on cell viability in a concentration-dependent fashion (**Figure 1C**). Vice versa, the inhibitory effect of ATO on viability of LOVO cells was also markedly enhanced by addition of AA in dose-dependent manner (**Figure 1D**). Qualitatively the same results were consistently observed with either ATO or AA alone or combination of the two (**Figures 1E–H**). These findings indicate the mutual facilitating effects or synergistic actions between ATO and AA in terms of their ability to induce cytotoxicity to CRC cells. In order to further confirm the synergistic actions of ATO combined with AA, we conducted CI analysis. Dose–effect analysis of the combination treatment was carried out based on the method of Chou-Talalay and the type of interaction between the two agents was evaluated using the CI (Chou, 2006). According to the predictive calculation based on the CompuSyn software, the results revealed that both ATO and AA killed LOVO cells in a concentration-dependent manner, with the half inhibitory doses (IC50) for ATO and AA being 11.4 μM and 7.7 mM for 24 h of incubation, respectively. Then low and non-toxic doses of AA (2 mM) were selected to be tested with various doses of ATO in the combination treatments. As shown in **Table 1**, the calculated CI values obtained were 0.44–0.53 for LOVO cells at 40–53% fractions affected. In addition, ATO concentration fixed at 2 μM in combination with increasing concentrations of AA for 24 h was used on LOVO cells. The results demonstrated that synergy between ATO and AA is moderate with CI = 0.43–0.66 for fa = 0.2–0.46 (**Table 1**). Additionally, the IC50 values in SW620 cells treated with ATO for 24 h was 13.3 μM and those of AA was 24.7 mM based on the predictive calculation in the CompuSyn software. The values of CI for combination treatment calculated in **Table 2** indicated that the significantly synergetic anti-cancer activity for combination treatment of AA and ATO in SW620 cells. To investigate the mechanism of the synergistic effect, we selected the combinations with 2 mM AA and 2 μM ATO in SW620 cells.

**Table 1:** Result of combination index analysis showing fraction affected and CI value in LOVO cells.

ATO (μM）	AA (mM)	Fa	CI	AA (mM)	ATO (μM）	Fa	CI
2	0.25	0.21	0.49	2	1	0.4	0.53
2	0.38	0.22	0.55	2	1.5	0.46	0.45
2	0.5	0.24	0.55	2	2	0.48	0.47
2	0.75	0.29	0.54	2	2.5	0.53	0.44
2	1	0.39	0.43				
2	1.5	0.41	0.5				
2	2	0.43	0.57				
2	3	0.46	0.66				

The CI values represent mean of three experiments. CI 0.8–0.9: slight synergism; CI 0.6–0.8: moderate synergism; CI 0.4–0.6: synergism; and CI 0.2–0.4: strong synergism. Fa, fraction affect; CI, combination index.

**Table 2:** Result of combination index analysis showing fraction affected and CI value in SW620 cells.

ATO (μM）	AA (mM)	Fa	CI	AA (mM）	ATO (μM）	Fa	CI
2	2	0.37	0.48	2	2	0.34	0.58
2	3	0.59	0.17	2	2.5	0.35	0.59

The CI values represent mean of three experiments. CI 0.8–0.9: slight synergism; CI 0.6–0.8: moderate synergism; CI 0.4–0.6: synergism; and CI 0.2–0.4: strong synergism. Fa, fraction affect; CI, combination index.

### ATO/AA Combination Decreases CRC Viability Partly by Inducing Apoptosis

**Figure 2:**
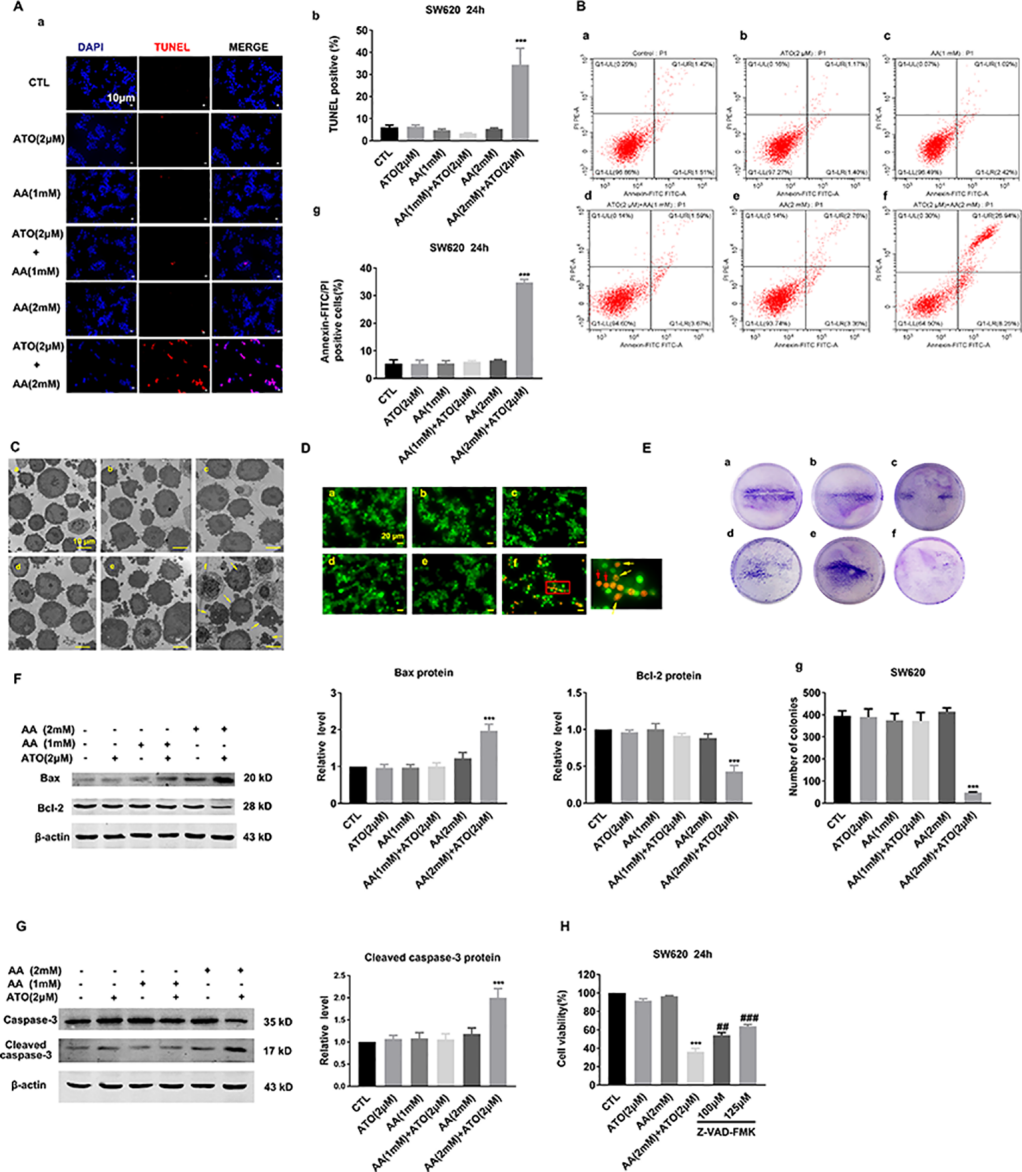
Effects of the co-treatment of AA and ATO on apoptosis of SW620 cells. **(A)** (a) Apoptotic cells were determined by TdT-mediated dUTP nick-end labeling (TUNEL) fluorescence staining of SW620 cells. The nuclei were stained blue with DAPI. Blue: DAPI; red: TUNEL. Magnification: ×200. Scale bar: 10 μm. (b) Statistical data of percentages of the TUNEL-positive cells counted from randomly selected fields of each images. ***P < 0.001 vs. control (Ctl). **(B)** Annexin V–FITC/PI staining for apoptosis in SW620 cells treated with ATO and AA. (a) Control; (b) ATO (2 μM); (c) AA (1 mM); (d) AA (1 mM) + ATO (2 μM); (e) AA (2 mM); (f) AA (2 mM) + ATO (2 μM). (g) Analysis results of representative flow cytometry images of SW620 cells apoptosis labeled with Annexin V–FITC/PI positive. All values represent the means with bars as SEM of three samples in an independent experiment, which were repeated three times with similar results.***P < 0.001 vs. Ctl. **(C)** Representative morphological changes of apoptotic cells determined by electron microscopic analysis. (a) control; (b) ATO (2 μM); (c) AA (1 mM); (d) AA (1 mM) + ATO (2 μM); (e) AA (2 mM); (f) AA (2 mM) + ATO (2 μM). The apoptosis cell was present with plasma and nuclear membrane blebbing and cytoplasmic vacuolization. Yellow arrows indicate the apoptosis cells. Magnification: ×2500. Scale bar: 10 μm. **(D)** Apoptotic cells were detected with Acridine orange/ethidium bromide (AO/EB) staining. (a) control; (b) ATO (2 μM); (c) AA (1 mM); (d) AA (1 mM) + ATO (2 μM); (e) AA (2 mM); (f) AA (2 mM) + ATO (2 μM). Yellow arrows indicate the apoptosis cells with nuclear collapse and chromatin condensed in lumps. Red arrows indicate the dead cells, such as necrosis and pyroptosis. Magnification: ×800. Scale bar: 20 μm. **(E)** Representative images of clonogenic assays after treatment with ATO and AA. (a) control; (b) ATO (2 μM); (c) AA (1 mM); (d) AA (1 mM) + ATO (2 μM); (e) AA (2 mM); (f) AA (2 mM) + ATO (2 μM). (g) Quantification of the clonogenic assay expressed as total number of surviving colonies. SW620 cells treated with ATO (2 μM) and AA (2 mM) had a significant decrease in clonogenic survival numbers compared with control. Viable colonies with diameter >0.3 mm were counted. Number of colonies in the resulting samples were quantified using the Image J analytical program. Data are expressed as the means ± SEM. All experiments were repeated at three times. ***P < 0.001 vs. Ctl. **(F)** Synergistic effects of ATO/AA combination (2 μM/1 mM or 2 mM) on the protein levels of pro-apoptotic Bax and anti-apoptotic Bcl-2 in SW620 cells determined by Western blot analysis. Left panel shows the representative Western blot bands and the right one presents percentage of cells with TUNEL-positive staining averaged from five independent (mean ± SEM normalized to β-actin as a loading control). ***P < 0.001 vs. Ctl. **(G)** Synergistic promotion of caspase-3 activation by ATO/AA combination, as indicated by robust increased protein levels of cleaved-caspase-3 in SW620 cells. β-Actin served as an internal control. Each bar represents mean ± SEM of five separate experiments. ***P < 0.001 vs. Ctl. **(H)** Countering effect of Z-VAD-FMK (pan-caspase inhibitor) on the suppression of viability of SW620 cells induced by ATO/AA combination. Data are presented as mean ± SEM of three independent experiments. ***P < 0.001 vs. Ctl. ^##^ *P <*0.01 vs. AA (2 mM) + ATO (2 μM). ^###^ *P* < 0.001 vs. AA (2 mM) + ATO (2 μM).

To elucidate whether the observed decreases in cell viability by ATO and AA are attributable to apoptotic cell death, we employed TUNEL staining to quantify DNA fragmentation. As depicted in **Figure 2A**, neither 2 μM ATO nor 2 mM AA induced apoptosis. However, the combination of the two compounds produced a remarkable pro-apoptotic effect. As depicted in **Figure 2B**, the single drug treatment did not increase the proportion of early and late apoptosis in SW620 cells. Furthermore, the combination of 2 μM ATO and 2 mM AA was found to more potently induce apoptosis in comparison to the single treatment. Similar results were obtained by morphological examination under an electron microscope (**Figure 2C**). Moreover, AO/EB staining indicated that apoptosis was markedly promoted in the combination of 2 μM ATO and 2 mM AA treated cells in compare with the single drug ATO or AA treatment (**Figure 2D**). Moreover, clonogenic assay demonstrated that ATO (2 μM) and AA (2 mM) combined application can significantly suppress the colony-forming activity of SW620 cells (**Figure 2E**).

To further elaborate whether the apoptotic cell death induced by ATO and AA is mediated by the mitochondrial death pathway, we determined the expression of anti-apoptotic protein Bcl-2 and pro-apoptotic protein Bax using Western blot analysis. As illustrated in **Figure 2F**, 2 μM ATO or 2 mM AA produced minimal effects on the protein levels of Bcl-2 and Bax. However, when co-applied, ATO (2 μM) and AA (2 mM) caused a substantial upregulation of Bax and concordant downregulation of Bcl-2 in SW620 cells. Moreover, the combination also synergistically increased the level of the cleaved or activated form of caspase-3, the executioner of apoptotic cell death (**Figure 2G**). Furthermore, exposure of SW620 cells to Z-VAD-FMK (a pan-caspase inhibitor) abrogated the suppressive effect of ATO/AA combination on cell viability (**Figure 2H**).

### ATO/AA Combination Decreases CRC Viability Partly by Inducing Pyroptosis

Pyroptosis is recognized as a new type of inflammatory programed cell-death, which is typically initiated by the binding of intracellular pathogen to NOD-like receptors (NLRs), leading to the formation of inflammasomes. Inflammasomes can subsequently cause release of the active forms of the pro-inflammatory cytokines IL-1β and IL-18 to activate caspase-1 which executes cell death (Miao et al., 2010).

**Figure 3:**
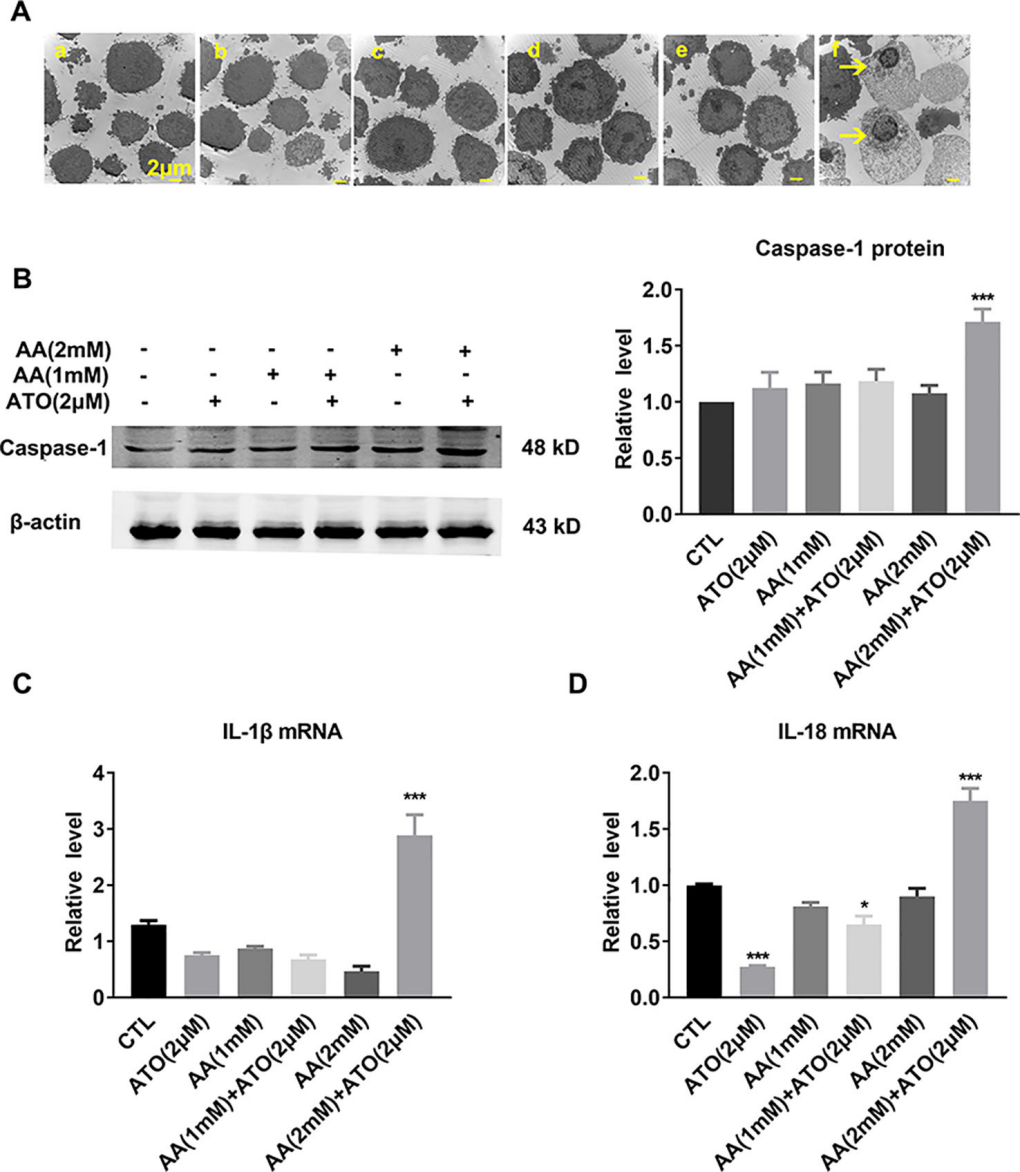
Effects of the co-treatment of AA and ATO on pyroptosis of SW620 cells. **(A)** Representative images of electron microscopic examination showing the promoting effects of ATO/AA combination treatment on pyroptosis. Yellow arrows indicate the pyroptotic cells with cytoplasmic swelling and nucleus pyknosis. (a) control; (b) ATO (2 μM); (c) AA (1 mM); (d) AA (1 mM) + ATO (2 μM); (e) AA (2 mM); (f) AA (2 mM) + ATO (2 μM). Magnification: ×10000. Scale bar: 2 μm. **(B)** Synergistic upregulation of caspase-1 protein level by ATO/AA combination treatment, with left panel showing the typical examples of Western blot band images and the right one showing the statistical data expressed as mean ± SEM. ***P < 0.001. **(C, D)** Synergistic elevation of the mRNA levels pro-inflammatory cytokines IL-1β and IL-18, respectively, by ATO/AA combination treatment in SW620 cells. *P < 0.05, ***P < 0.001. The data are presented as mean ± SEM (n = 3).

The electron microscope images shown in **Figure 3A** displayed the characteristic morphological features of pyroptosis, as evidenced by swelling and nuclear shrinkage of SW620 cells after treatment with ATO/AA combination, but not with ATO or AA alone. Consistently, the level of caspase-1 protein was significantly increased by the combination (**Figure 3B**). Meanwhile, the mRNA levels of pro-inflammatory cytokines IL-1β and IL-18, the pyroptosis-related genes, were also significantly upregulated by ATO and AA combination (**Figures 3C, D**).

### ATO/AA Combination Synergistically Produces Pro-Apoptotic Effects by Stimulating ROS Generation

**Figure 4:**
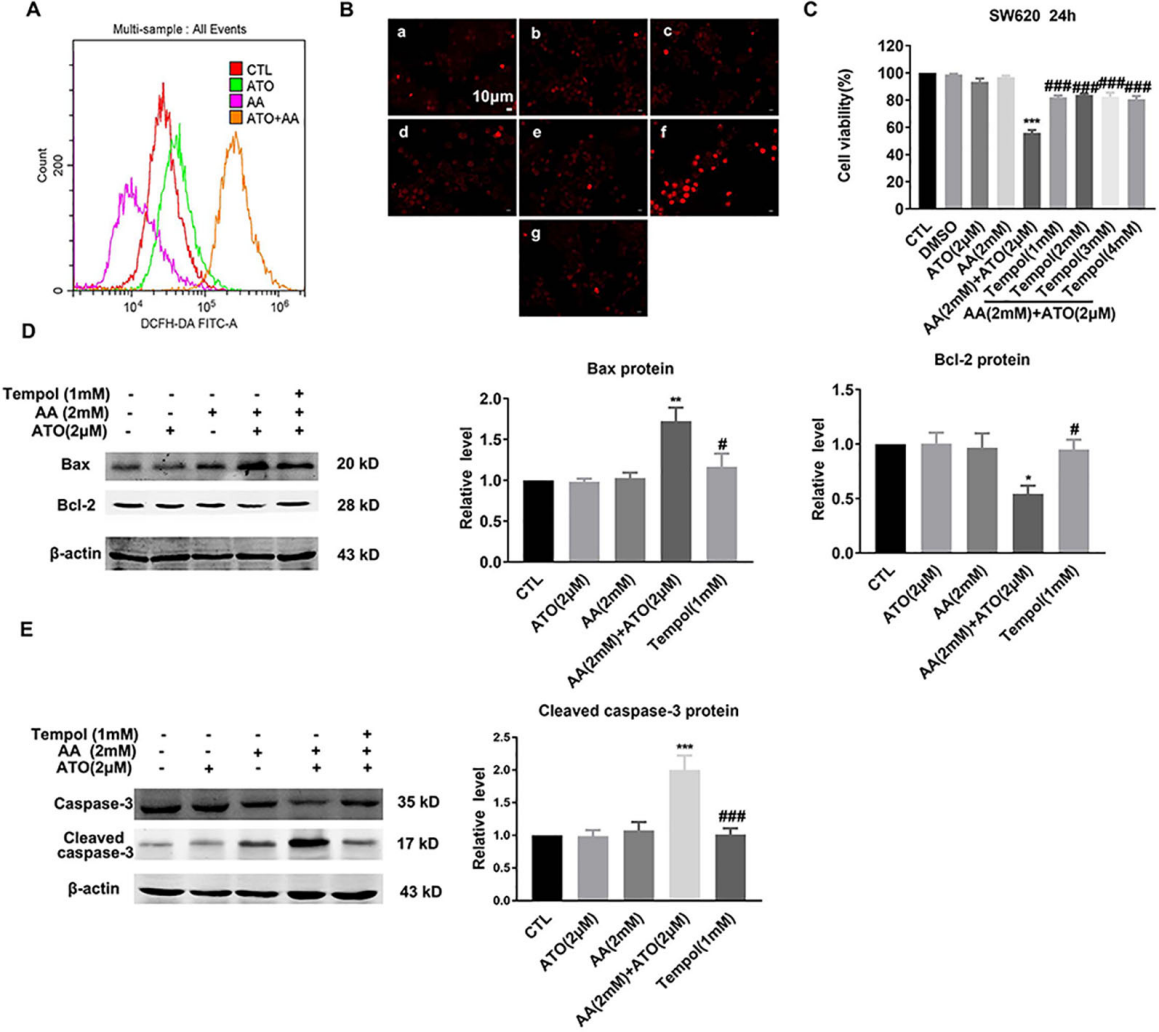
Antagonistic effects of tempol on the pro-apoptotic action of ATO (2 μM)/AA (2 mM) combination in SW620 cells. **(A)** Effects of ATO, AA, and ATO/AA combination on the levels of intracellular reactive oxygen species (ROS) as measured by flow cytometry using an oxidation-sensitive fluorescent probe, 2′,7′-dichlorodihydrofluorescein diacetate (DCFH-DA). Note that ATO/AA combination caused the greatest ROS production as indicated by the highest cell count of DCFH-DA staining. **(B)** Inhibitory effect of an ROS scavenger tempol (1 mM) on the synergistic enhancement of ROS production induced by ATO (2 μM)/AA (2 mM) combination treatment of SW620 cells, as determined by a ROS fluorescent probe. (a) Control, (b) ATO (2 μM); (c) AA (1 mM); (d) AA (1 mM) + ATO (2 μM); (e) AA (2 mM); (f) AA (2 mM) + ATO (2 μM); (g) AA (2 mM) + ATO (2 μM) + tempol (1 mM). Magnification: ×200. Scale bar: 10 μm. **(C)** Inhibitory effect of tempol (1 mM) on the synergistic suppression of cell viability induced by ATO (2 μM)/AA (2 mM) combination treatment of SW620 cells. SW620 cells were pretreated with tempol for 1 h before incubating with ATO (2 μM) + AA (2 mM) for 24 h. The data are represented as mean ± SEM (n = 3). ***P < 0.001 vs. Ctl. ^###^ *P* < 0.001 vs. AA + ATO. **(D)** Alleviating effects of tempol on the upregulation of Bax protein and downregulation of Bcl-2 protein induced by ATO (2 μM)/AA (2 mM) combination treatment of SW620 cells. **(E)** Reversing effect of tempol on upregulation of cleaved-caspase-3 levels induced by ATO (2 μM)/AA (2 mM) combination treatment. Beta-actin was used as an internal control. **P < 0.01, vs. Ctl. ^#^ *P* < 0.05 vs. AA and ATO. ^##^ *P* < 0.01 vs. AA +ATO; n = 5.

Previous studies have established that ROS produced through the oxidation of AA is a crucial mediator of AA-induced cytotoxicity (Gong et al., 2016). Similarly, ROS production to induce oxidative stress is also the primary mechanism by which ATO causes apoptosis of tumor cells (Baysan et al., 2007). To elaborate if the observed synergistic effects of ATO and AA in inducing apoptosis of CRC cells could be ascribed to their ability to stimulate ROS production and accumulation, we measured the changes of ROS contents under varying conditions. As illustrated in **Figure 4A**, AA/ATO combination increases ROS levels by flow cytometry. Similar results were obtained with fluorescence staining of ROS (**Figure 4B**). Tempol, which is a stable piperidine nitroxide of low molecular weight, can easily permeates biological membranes and accumulates in the cytosol. Tempol could act as an intracellular scavenger of superoxide anions, particularly, hydroxyl radicals (Wilcox, 2010). Tempol specifically targets mitochondrial ROS. Mariappan *et al*. demonstrated that tempol prevented O2^−^ production by mitochondrial complex 1 when tumor necrosis factor alpha was administered (Mariappan et al., 2009). It has also been shown to prevent angiotensin II from stimulating mitochondrial ROS (Kimura et al., 2005). In addition, the suppressing effects of ATO and AA on cell viability was significantly mitigated by tempol, a ROS inhibitor (**Figure 4C**). Consistently, tempol also prevented the upregulation of Bax and downregulation of Bcl-2 induced by ATO/AA combination (**Figure 4D**), as well as the increase in cleaved caspase-3 level in SW620 cells (**Figure 4E**).

### ATO/AA Combination Synergistically Produces Pro-Pyroptotic Effects by Stimulating ROS Generation

**Figure 5:**
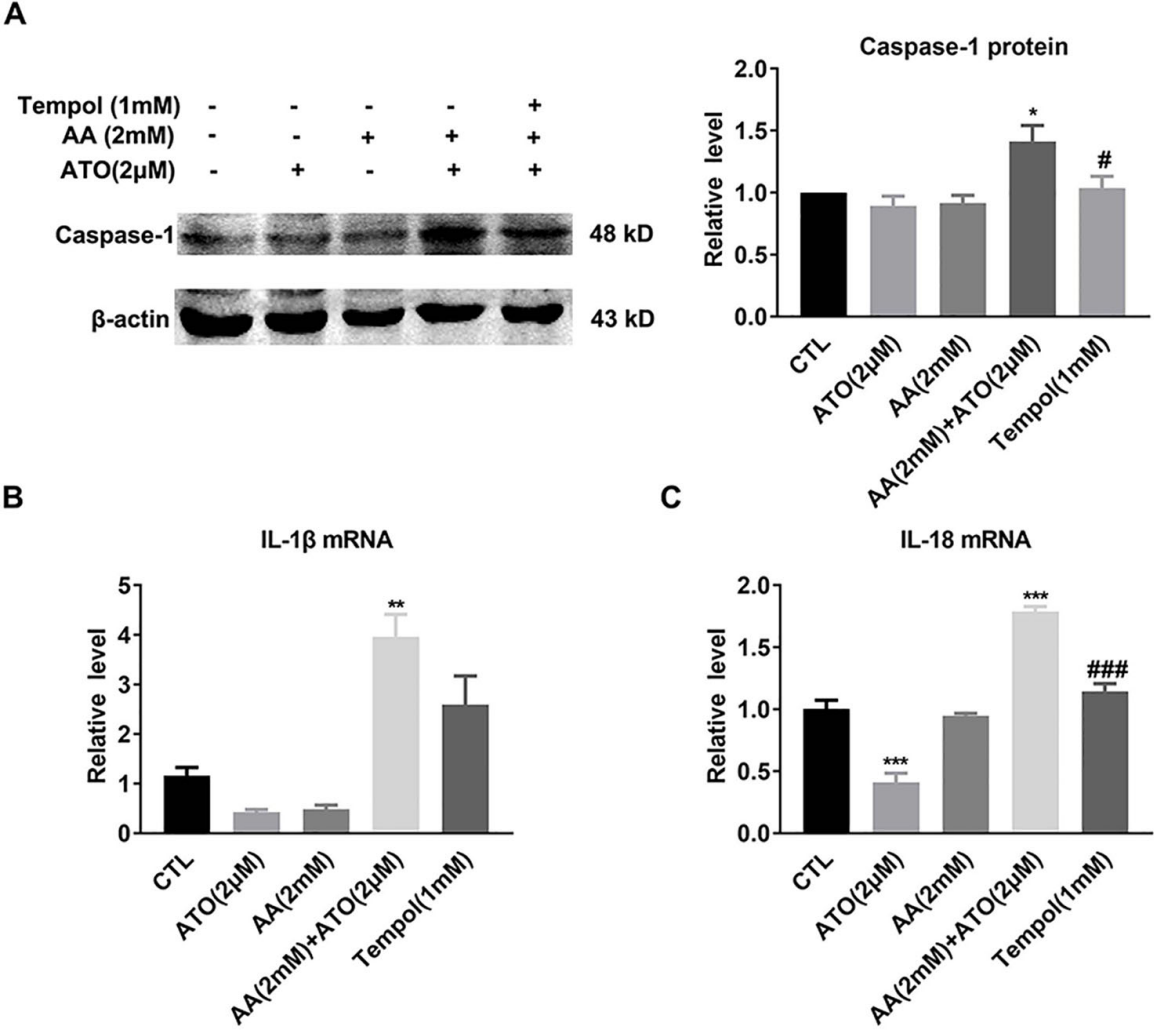
Antagonistic effects of tempol on the pro-pyroptotic action of ATO (2 μM)/AA (2 mM) combination in SW620 cells. **(A)** Tempol (1 mM) abolishes the elevation of caspase-1 protein level induced by ATO (2 μM)/AA (2 mM) combination treatment. The data are represented as mean ± SEM (n = 5). *P < 0.05, vs. Ctl; ^#^ *P* < 0.05 vs. AA + ATO. **(B, C)** Tempol (1 mM) mitigated the increased mRNA levels of IL-1β and IL-18 induced by ATO/AA combination treatment. The data shown are mean ± SEM of three independent experiments. **P < 0.01, vs. Ctl. ***P < 0.001 vs. Ctl; ^###^ *P* < 0.001 vs. AA + ATO.

As already described above, ATO/AA combination synergistically elevated the protein level of caspase-1 and the mRNA levels of IL-1β and IL-18 as well (**Figures 3B–D**). In order to verify that ROS underlies at least partly the observed effects, we went on to evaluate the influence of tempol on the effects of ATO/AA combination. As depicted in **Figure 5**, exposure of SW620 cells to tempol pronouncedly abrogated the upregulation of caspase-1 protein (**Figure 5A**) and IL-1β and IL-18 mRNAs induced by ATO/AA combination (**Figures 5B, C**).

## Discussion

The findings of our present study indicate that the synergistic interaction between AA and ATO in CRC cells. AA significantly potentiates the effect of ATO on cell death of CRC and vice versa. This interaction facilitates ROS production in CRC cells to induce apoptosis and pyroptosis.

ATO has been clinically recommended as a potent anti-leukemic agent for clinical therapy of complete remission in patients with refractory acute promyelocytic leukemia, a M3 subtype of AML (Soignet et al., 1998). At clinically achievable concentrations of 1–2 μM ATO induces apoptosis in patients with APL with minimal side effects (Chen et al., 1996). The cytotoxic effect of ATO on APL is thought to be mediated by its ability to induce oxidative stress. The median daily dose of ATO in a pilot study was 0.15 mg/kg (0.06–0.2 mg/kg), within the clinically relevant concentrations being 0.1–1.5 mM (Siu et al., 2006).

We have previously demonstrated that only a high dose of ATO (> 2 μM) directly increases ROS production and induces apoptosis in myocytes (Zhao et al., 2008). Overwhelming reports have suggested that ATO is not limited to the treatment of leukemia, but has also the potential to suppress solid tumors such as breast cancer, hepatocellular carcinoma, and CRC (Rollig and Illmer, 2009; Wang et al., 2014; Eyvani et al., 2016; Yoon et al., 2016; Shi et al., 2017). The mechanism of ATO-induced apoptosis is mainly ascribed to ROS over-generation. Nevertheless, ATO can cause severe toxicities such as long QT syndrome/torsade de pointes and sudden cardiac death, which hampers the clinical applications of ATO to solid tumors (Li et al., 2013).

On the other hand, clinical efficiency of anticancer drugs is frequently limited by emergence of various mechanisms of resistance in tumor cells (Vernhet et al., 2001), and ATO has also been associated with a rapid development of clinical resistance in patients (Soignet et al., 1998). In 2007, Subbarayan et al. conducted a clinical trial to assess the efficacy and toxicity of ATO in patients with refractory metastatic colorectal carcinoma (CRC). Under the treatment regimen all patients developed moderate to severe side effects with no clinically measurable activity (Subbarayan et al., 2007).

In general, side effects and drug resistance due to high-dose applications partly account for the failure of single ATO treatment in solid tumors. Clearly, avoiding toxicity and eliminating drug resistance of ATO are critical and highly desirable for the applications of ATO to curing solid tumors. Given most of the solid tumors are believed to be hypoxic, solid tumors do not respond to arsenic. In addition, inner core of tumors lack blood vessels and therefore may not receive ATO. Thus, non-delivery of ATO to solid tumors may be a reason for the treatment failure (Subbarayan et al., 2007). One way to tackle the issues is using conventional doses of ATO while keeping high therapeutic efficacy by adopting the approach of combinational drug applications.

AA functions primarily as an antioxidant and can protect cells from oxidative stress at lower concentrations. However, at higher concentrations AA acts as a pro-oxidant that imposes oxidative stress and induces cell death; It has been proposed to have cancer therapeutic potential (Verrax and Calderon, 2009). Chen et al. revealed that when administered at high doses in the millimolar (mM) range, AA behaves as a powerful pro-oxidant, generating hydrogen peroxide-dependent cytotoxicity toward a variety of cancer cells *in vitro* (Chen et al., 2007). On the other hand, ATO is known to be a pro-oxidant *via* downregulating ROS scavenger proteins and disrupting redox pathways. We reasoned that ATO could interact with AA and produce synergistic effects against tumors. Indeed, AA has been shown to synergize the cytotoxic effects of ATO in several types of cancers, such as B-cell chronic lymphocytic leukemia, lymphoid malignancies, multiple myeloma, and pancreatic cancer (Chang et al., 2009; Biswas et al., 2010; Dinnen et al., 2013). Yet, the cytotoxicity effect of AA and ATO combination on colon cancer remains largely obscure. In the present study, we confirmed that AA could reinforce ATO-induced apoptosis by promoting ROS overproduction. Moreover, some clinicians have infused more than 10 g of ascorbate in cancer patients and achieved plasma concentrations of 1 to 5 mM, which showed survival benefit and symptomatic relief (Riordan et al., 1995). In our research, we conducted 2 mM concentration of AA co-treated with 2 μM ATO, which mostly kills CRC cells by inducing cyto-apoptosis and pyroptosis *via* increasing intracellular ROS. In addition, vitamin C accumulates in solid tumors to concentrations higher than in surrounding normal tissue (Agus et al., 1999). This phenomenon favors the positive outcome of high-dose intravenous vitamin C therapy in cancer patients. So our study may have clinical meaning for the survival benefit and symptomatic relief of cancer patients.

Pyroptosis is a form of lytic programmed cell death initiated by inflammasomes that activate caspase-1 followed by releasing pro-inflammatory factors IL-1β and IL-18. We show here for the first time that AA and ATO combination synergistically enhanced pyroptosis and the underlying mechanism is likely to promote overproduction and accumulation of intracellular ROS, a critical inducer of the inflammasome-dependent pyroptosis (Zhou et al., 2018). Interestingly, our results demonstrate that the combination of ATO and AA synergistically enhanced pyroptosis by stimulating ROS generation and upregulated the expression levels of IL-1beta and IL-18 mRNAs, whereas each of the two drugs downregulated the expression of the two cytokines (**Figures 3C, D**; **5B, C**). The combination of ATO and AA synergistically upregulated the expression levels of IL-1beta and IL-18 mRNAs which were pro-inflammatory factors released by pyroptosis. Maier et al. indicated that ATO inhibited activation of the nucleotide-binding oligomerization domain-like receptor (NLR) proteins inflammasomes, preventing the autoproteolytic activation of caspase-1 and the processing and secretion of IL-1β from macrophages (Maier et al., 2014). And several studies revealed that ATO markedly reduced IL-18, NO, TNF-α, and IL-10 levels in MRL/lpr mice developing human lupus-like syndrome and inhibited NF-κB expression and DNA binding in colon extracts, leading to decreased cytokine gene expression (e.g., TNF-α, IL-1β, IL-17, and IL-18) in a murine colitis model (Bobe et al., 2006; Singer et al., 2011). However, ATO was found to induce apoptosis in several cancer cells *via* inducing ROS production. In agreement with previous results, the accumulation of ROS production may contribute to the upregulation of IL-1beta and IL-8 mRNAs. So we still need to explore whether there exists other mechanisms underlying that lead to the downregulation of the expression of the two cytokines by adding ATO/AA.

Collectively, our results provide the first evidence that AA could sensitize ATO-induced pyroptosis by stimulating ROS production. Hence, pyroptosis is likely one of the cellular mechanisms underlying the cytotoxicity effect of AA and ATO on CRC. The role of ROS in mediating the enhanced cytotoxicity (apoptosis and pyroptosis) of AA/ATO combination against CRC cells was verified by the results exhibiting the effectiveness of ROS scavenger Tempol in mitigating the cytotoxicity of AA/ATO co-application.

**Figure 6:**
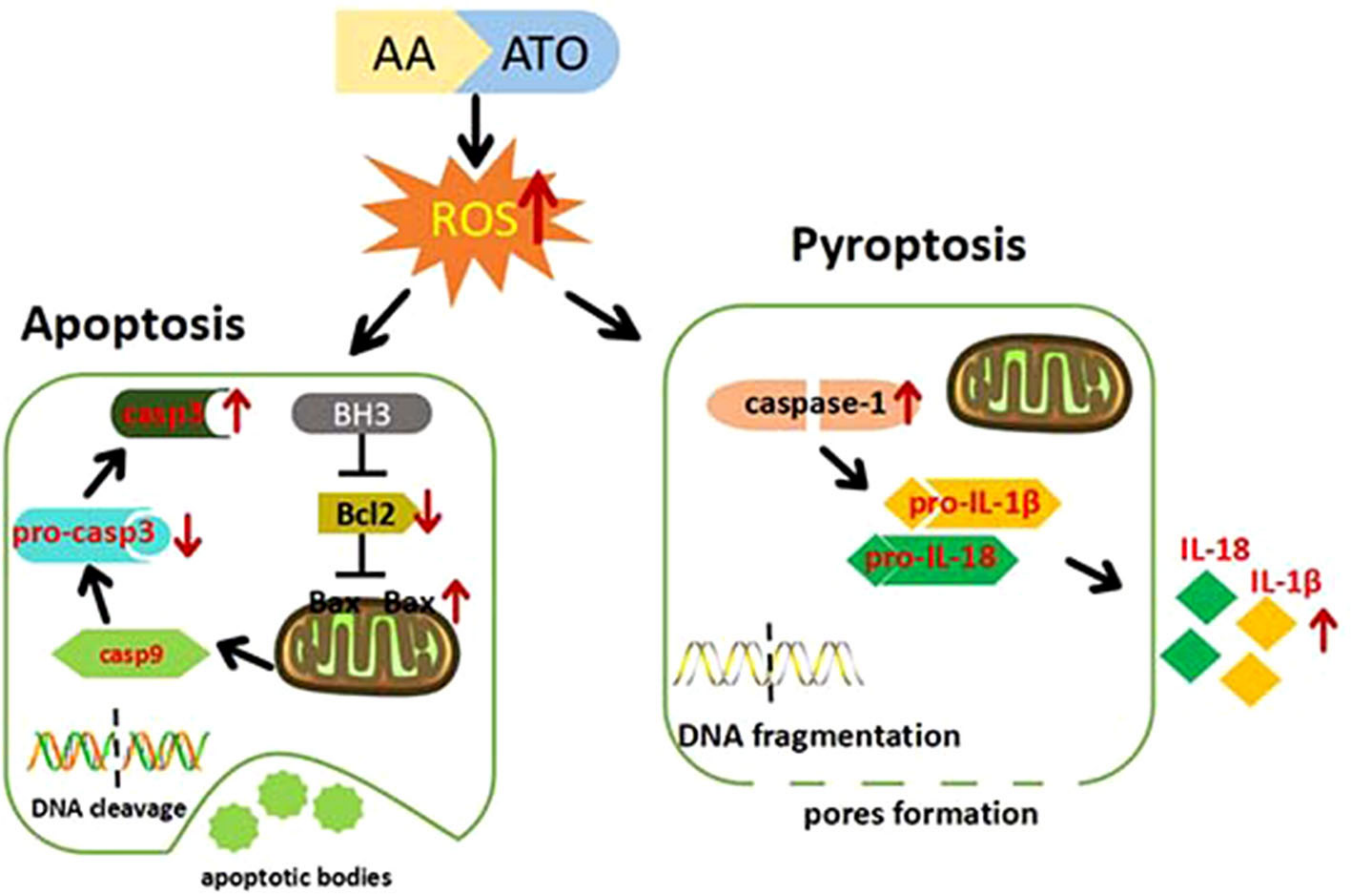
Cartoon presentation of proposed cellular and molecular mechanisms for the synergistic effects of ATO/AA combination treatment. AA and ATO enter the colorectal cancer cells and induce overproduction of ROS that in turn activates the mitochondrial apoptotic pathway with increased expression of Bax and caspase-3 and decreased expression of Bcl-2. On the other hand, AA and ATO activate pyroptosis signaling by increasing caspase-1 and releasing IL-1β and IL-18. AA sensitizes ATO-induced apoptosis and pyroptosis by ROS-dependent oxidative stress in colon cancer cells.

Collectively, we present the evidence for the superiority of ATO/AA combination with a conventional dosage of ATO and AA for cancer therapy over ATO or AA alone at higher doses in the context of generating cytotoxicity against CRC cells. We also demonstrate that enhanced apoptosis and pyroptosis are likely a cellular mechanism for the synergistic action of ATO/AA combination. Furthermore, we provide evidence for ROS overproduction as a molecular/metabolic mechanism for the induction of apoptosis and pyroptosis by ATO/AA combination treatment (**Figure 6**). Our findings therefore provide a rationale for ATO/AA combination as a plausible strategy for the treatment of CRC and other solid tumors as well.

## Data Availability Statement

All datasets generated for this study are included in the article/supplementary material.

## Author Contributions

B-FY and W-FC designed the experiments and supervised the project. WT was responsible for the manuscript writing and data analysis. YL revised the manuscript. J-TL, ZW, and N-NT performed most experiments.

## Funding

This study was supported by the National Natural Science Foundation of China (31701021, 81770281, and 81730012) and Transformation Achievement Cultivation Project of Harbin Medical University (2018010316).

## Conflict of Interest

The authors declare that the research was conducted in the absence of any commercial or financial relationships that could be construed as a potential conflict of interest.

## Abbreviations

AA, ascorbic acid; AML, acute myeloid leukemia; APL, acute promyelocytic leukemia; ATO, arsenic trioxide; CCK-8, Cell Counting Kit-8; Ctl, control; CRC, colorectal cancer; DCF, 2,7-dichlorofluorescein; DCFH-DA, 2′,7′-dichlorodihydrofluorescein diacetate; FBS, fetal bovine serum; GSDMD, gasdermin D; IL, interleukin; NLRs, NOD-like receptors; PBS, phosphate-buffered saline; ROS, reactive oxygen species; TUNEL, TdT-mediated dUTP nick-end labeling.

## References

[B1] Agus D. B. Vera J. C. Golde D. W. (1999). Stromal cell oxidation: a mechanism by which tumors obtain vitamin C. Cancer Res. 59 (18), 4555–4558. 10493506

[B2] Arnold M. Sierra M. S. Laversanne M. Soerjomataram I. Jemal A. Bray F. (2017). Global patterns and trends in colorectal cancer incidence and mortality. Gut 66 (4), 683–691. 10.1136/gutjnl-2015-310912 26818619

[B3] Baysan A. Yel L. Gollapudi S. Su H. Gupta S. (2007). Arsenic trioxide induces apoptosis *via the* mitochondrial pathway by upregulating the expression of Bax and Bim in human B cells. Int. J. Oncol. 30 (2), 313–318. 10.3892/ijo.30.2.313 17203211

[B4] Biswas S. Zhao X. Mone A. P. Mo X. Vargo M. Jarjoura D. (2010). Arsenic trioxide and ascorbic acid demonstrate promising activity against primary human CLL cells *in vitro* . Leuk Res. 34 (7), 925–931. 10.1016/j.leukres.2010.01.020 20171736 PMC4164821

[B5] Bobe P. Bonardelle D. Benihoud K. Opolon P. Chelbi-Alix M. K. (2006). Arsenic trioxide: a promising novel therapeutic agent for lymphoproliferative and autoimmune syndromes in MRL/lpr mice. Blood 108 (13), 3967–3975. 10.1182/blood-2006-04-020610 16926289

[B6] Cameron E. Pauling L. (1976). Supplemental ascorbate in the supportive treatment of cancer: Prolongation of survival times in terminal human cancer. Proc. Natl. Acad. Sci. U. S. A 73 (10), 3685–3689. 10.1073/pnas.73.10.3685 1068480 PMC431183

[B7] Chang J. E. Voorhees P. M. Kolesar J. M. Ahuja H. G. Sanchez F. A. Rodriguez G. A. (2009). Phase II study of arsenic trioxide and ascorbic acid for relapsed or refractory lymphoid malignancies: a wisconsin oncology network study. Hematol. Oncol. 27 (1), 11–16. 10.1002/hon.870 PMC2897137 18668698

[B8] Chen G. Y. Nunez G. (2011). Inflammasomes in intestinal inflammation and cancer. Gastroenterology 141 (6), 1986–1999. 10.1053/j.gastro.2011.10.002 22005480 PMC3442608

[B9] Chen G. Q. Zhu J. Shi X. G. Ni J. H. Zhong H. J. Si G. Y. (1996). In vitro studies on cellular and molecular mechanisms of arsenic trioxide (As2O3) in the treatment of acute promyelocytic leukemia: As2O3 induces NB4 cell apoptosis with downregulation of Bcl-2 expression and modulation of PML-RAR alpha/PML proteins. Blood 88 (3), 1052–1061. 10.1182/blood.V88.3.1052.1052 8704214

[B10] Chen Q. Espey M. G. Sun A. Y. Lee J. H. Krishna M. C. Shacter E. (2007). Ascorbate in pharmacologic concentrations selectively generates ascorbate radical and hydrogen peroxide in extracellular fluid *in vivo* . Proc. Natl. Acad. Sci. U. S. A. 104 (21), 8749–8754. 10.1073/pnas.0702854104 17502596 PMC1885574

[B11] Chen X. Dai X. Zou P. Chen W. Rajamanickam V. Feng C. (2017). Curcuminoid EF24 enhances the anti-tumour activity of Akt inhibitor MK-2206 through ROS-mediated endoplasmic reticulum stress and mitochondrial dysfunction in gastric cancer. Br. J. Pharmacol. 174 (10), 1131–1146. 10.1111/bph.13765 28255993 PMC5406301

[B12] Chou T. C. (2006). Theoretical basis, experimental design, and computerized simulation of synergism and antagonism in drug combination studies. Pharmacol. Rev. 58 (3), 621–681. 10.1124/pr.58.3.10 16968952

[B13] Chu W. Li C. Qu X. Zhao D. Wang X. Yu X. (2012). Arsenic-induced interstitial myocardial fibrosis reveals a new insight into drug-induced long QT syndrome. Cardiovasc. Res. 96 (1), 90–98. 10.1093/cvr/cvs230 22853924

[B14] De Laurenzi V. Melino G. Savini I. Annicchiarico-Petruzzelli M. Finazzi-Agro A. Avigliano L. (1995). Cell death by oxidative stress and ascorbic acid regeneration in human neuroectodermal cell lines. Eur. J. Cancer 31A (4), 463–466. 10.1016/0959-8049(95)00059-R 7576946

[B15] Derangere V. Chevriaux A. Courtaut F. Bruchard M. Berger H. Chalmin F. (2014). Liver X receptor beta activation induces pyroptosis of human and murine colon cancer cells. Cell Death Differ 21 (12), 1914–1924. 10.1038/cdd.2014.117 25124554 PMC4227150

[B16] Dinnen R. D. Mao Y. Qiu W. Cassai N. Slavkovich V. N. Nichols G. (2013). Redirecting apoptosis to aponecrosis induces selective cytotoxicity to pancreatic cancer cells through increased ROS, decline in ATP levels, and VDAC. Mol. Cancer Ther. 12 (12), 2792–2803. 10.1158/1535-7163.MCT-13-0234 24126434 PMC4318573

[B17] Eyvani H. Moghaddaskho F. Kabuli M. Zekri A. Momeny M. Tavakkoly-Bazzaz J. (2016). Arsenic trioxide induces cell cycle arrest and alters DNA methylation patterns of cell cycle regulatory genes in colorectal cancer cells. Life Sci. 167, 67–77. 10.1016/j.lfs.2016.10.020 27769816

[B18] Ghobrial I. M. Witzig T. E. Adjei A. A. (2005). Targeting apoptosis pathways in cancer therapy. CA Cancer J. Clin. 55 (3), 178–194. 10.3322/canjclin.55.3.178 15890640

[B19] Gong E. Y. Shin Y. J. Hwang I. Y. Kim J. H. Kim S. M. Moon J. H. (2016). Combined treatment with vitamin C and sulindac synergistically induces p53- and ROS-dependent apoptosis in human colon cancer cells. Toxicol. Lett. 258, 126–133. 10.1016/j.toxlet.2016.06.019 27339904

[B20] He W. T. Wan H. Hu L. Chen P. Wang X. Huang Z. (2015). Gasdermin D is an executor of pyroptosis and required for interleukin-1beta secretion. Cell Res. 25 (12), 1285–1298. 10.1038/cr.2015.139 26611636 PMC4670995

[B21] Hoffer L. J. Levine M. Assouline S. Melnychuk D. Padayatty S. J. Rosadiuk K. (2008). Phase I clinical trial of i.v. ascorbic acid in advanced malignancy. Ann. Oncol. 19 (11), 1969–1974. 10.1093/annonc/mdn377 18544557

[B22] Hughes M. F. (2002). Arsenic toxicity and potential mechanisms of action. Toxicol. Lett. 133 (1), 1–16. 10.1016/S0378-4274(02)00084-X 12076506

[B23] Kimura S. Zhang G. X. Nishiyama A. Shokoji T. Yao L. Fan Y. Y. (2005). Mitochondria-derived reactive oxygen species and vascular MAP kinases: comparison of angiotensin II and diazoxide. Hypertension 45 (3), 438–444. 10.1161/01.HYP.0000157169.27818.ae 15699441

[B24] Li C. Qu X. Xu W. Qu N. Mei L. Liu Y. (2013). Arsenic trioxide induces cardiac fibroblast apoptosis *in vitro* and *in vivo* by up-regulating TGF-beta1 expression. Toxicol. Lett. 219 (3), 223–230. 10.1016/j.toxlet.2013.03.024 23542815

[B25] Li R. Jia Z. Trush M. A. (2016). Defining ROS in biology and medicine. React Oxyg Species (Apex) 1 (1), 9–21. 10.20455/ros.2016.803 29707643 PMC5921829

[B26] Lin C. Zhang J. (2017). Inflammasomes in inflammation-induced cancer. Front. Immunol. 8, 271. 10.3389/fimmu.2017.00271 28360909 PMC5350111

[B27] Ma Y. Chapman J. Levine M. Polireddy K. Drisko J. Chen Q. (2014). High-dose parenteral ascorbate enhanced chemosensitivity of ovarian cancer and reduced toxicity of chemotherapy. Sci. Transl. Med. 6 (222), 222ra218. 10.1126/scitranslmed.3007154 24500406

[B28] Maier N. K. Crown D. Liu J. Leppla S. H. Moayeri M. (2014). Arsenic trioxide and other arsenical compounds inhibit the NLRP1, NLRP3, and NAIP5/NLRC4 inflammasomes. J. Immunol. 192 (2), 763–770. 10.4049/jimmunol.1301434 24337744 PMC3884817

[B29] Mandl J. Szarka A. Banhegyi G. (2009). Vitamin C: update on physiology and pharmacology. Br. J. Pharmacol. 157 (7), 1097–1110. 10.1111/j.1476-5381.2009.00282.x 19508394 PMC2743829

[B30] Mariappan N. Elks C. M. Fink B. Francis J. (2009). TNF-induced mitochondrial damage: a link between mitochondrial complex I activity and left ventricular dysfunction. Free Radic. Biol. Med. 46 (4), 462–470. 10.1016/j.freeradbiomed.2008.10.049 19041937 PMC2735225

[B31] Martin-Martin N. Piva M. Urosevic J. Aldaz P. Sutherland J. D. Fernandez-Ruiz S. (2016). Stratification and therapeutic potential of PML in metastatic breast cancer. Nat. Commun. 7, 12595. 10.1038/ncomms12595 27553708 PMC4999521

[B32] Mathews V. George B. Chendamarai E. Lakshmi K. M. Desire S. Balasubramanian P. (2010). Single-agent arsenic trioxide in the treatment of newly diagnosed acute promyelocytic leukemia: long-term follow-up data. J. Clin. Oncol. 28 (24), 3866–3871. 10.1200/JCO.2010.28.5031 20644086

[B33] McConnell M. J. Herst P. M. (2014). Ascorbate combination therapy: new tool in the anticancer toolbox? Sci. Transl. Med. 6 (222), 222fs226. 10.1126/scitranslmed.3008488 24500402

[B34] Miao E. A. Leaf I. A. Treuting P. M. Mao D. P. Dors M. Sarkar A. (2010). Caspase-1-induced pyroptosis is an innate immune effector mechanism against intracellular bacteria. Nat. Immunol. 11 (12), 1136–1142. 10.1038/ni.1960 21057511 PMC3058225

[B35] Moertel C. G. Fleming T. R. Creagan E. T. Rubin J. O’Connell M. J. Ames M. M. (1985). High-dose vitamin C versus placebo in the treatment of patients with advanced cancer who have had no prior chemotherapy. a randomized double-blind comparison. N Engl. J. Med. 312 (3), 137–141. 10.1056/NEJM198501173120301 3880867

[B36] Pettersson H. M. Pietras A. Munksgaard Persson M. Karlsson J. Johansson L. Shoshan M. C. (2009). Arsenic trioxide is highly cytotoxic to small cell lung carcinoma cells. Mol. Cancer Ther. 8 (1), 160–170. 10.1158/1535-7163.MCT-08-0595 19139125

[B37] Riordan N. H. Riordan H. D. Meng X. Li Y. Jackson J. A. (1995). Intravenous ascorbate as a tumor cytotoxic chemotherapeutic agent. Med. Hypotheses 44 (3), 207–213. 10.1016/0306-9877(95)90137-x 7609676

[B38] Rollig C. Illmer T. (2009). The efficacy of arsenic trioxide for the treatment of relapsed and refractory multiple myeloma: a systematic review. Cancer Treat Rev. 35 (5), 425–430. 10.1016/j.ctrv.2009.04.007 19464807

[B39] Schwartz J. L. (1996). The dual roles of nutrients as antioxidants and prooxidants: their effects on tumor cell growth. J. Nutr. 126 (4 Suppl), 1221S–1227S. 10.1093/jn/126.suppl_4.1221S 8642460

[B40] Shi Y. Cao T. Huang H. Lian C. Yang Y. Wang Z. (2017). Arsenic trioxide inhibits cell growth and motility *via* up-regulation of let-7a in breast cancer cells. Cell Cycle 16 (24), 2396–2403. 10.1080/15384101.2017.1387699 28980872 PMC5788432

[B41] Siegel R. L. Miller K. D. Jemal A. (2018). Cancer statistics 2018. CA Cancer J. Clin. 68 (1), 7–30. 10.3322/caac.21442 29313949

[B42] Singer M. Trugnan G. Chelbi-Alix M. K. (2011). Arsenic trioxide reduces 2,4,6-trinitrobenzene sulfonic acid-induced murine colitis *via* nuclear factor-kappaB down-regulation and caspase-3 activation. Innate Immun. 17 (4), 365–374. 10.1177/1753425910371668 20693187

[B43] Siu C. W. Au W. Y. Yung C. Kumana C. R. Lau C. P. Kwong Y. L. (2006). Effects of oral arsenic trioxide therapy on QT intervals in patients with acute promyelocytic leukemia: implications for long-term cardiac safety. Blood 108 (1), 103–106. 10.1182/blood-2006-01-0054 16514059

[B44] Soignet S. L. Maslak P. Wang Z. G. Jhanwar S. Calleja E. Dardashti L. J. (1998). Complete remission after treatment of acute promyelocytic leukemia with arsenic trioxide. N Engl. J. Med. 339 (19), 1341–1348. 10.1056/NEJM199811053391901 9801394

[B45] Stephenson C. M. Levin R. D. Spector T. Lis C. G. (2013). Phase I clinical trial to evaluate the safety, tolerability, and pharmacokinetics of high-dose intravenous ascorbic acid in patients with advanced cancer. Cancer Chemother. Pharmacol. 72 (1), 139–146. 10.1007/s00280-013-2179-9 23670640 PMC3691494

[B46] Subbarayan P. R. Ardalan B. (2014). In the war against solid tumors arsenic trioxide needs partners. J. Gastrointest Cancer 45 (3), 363–371. 10.1007/s12029-014-9617-8 24825822

[B47] Subbarayan P. R. Lima M. Ardalan B. (2007). Arsenic trioxide/ascorbic acid therapy in patients with refractory metastatic colorectal carcinoma: a clinical experience. Acta Oncol. 46 (4), 557–561. 10.1080/02841860601042456 17497326

[B48] Tan M. Zhang Q. Yuan X. Chen Y. Wu Y. (2019). Synergistic killing effects of homoharringtonine and arsenic trioxide on acute myeloid leukemia stem cells and the underlying mechanisms. J. Exp. Clin. Cancer Res. 38 (1), 308. 10.1186/s13046-019-1295-8 31307525 PMC6631946

[B49] Vernhet L. Allain N. Payen L. Anger J. P. Guillouzo A. Fardel O. (2001). Resistance of human multidrug resistance-associated protein 1-overexpressing lung tumor cells to the anticancer drug arsenic trioxide. Biochem. Pharmacol. 61 (11), 1387–1391. 10.1016/S0006-2952(01)00606-2 11331074

[B50] Verrax J. Calderon P. B. (2009). Pharmacologic concentrations of ascorbate are achieved by parenteral administration and exhibit antitumoral effects. Free Radic. Biol. Med. 47 (1), 32–40. 10.1016/j.freeradbiomed.2009.02.016 19254759

[B51] Vuyyuri S. B. Rinkinen J. Worden E. Shim H. Lee S. Davis K. R. (2013). Ascorbic acid and a cytostatic inhibitor of glycolysis synergistically induce apoptosis in non-small cell lung cancer cells. PloS One 8 (6), e67081. 10.1371/journal.pone.0067081 23776707 PMC3679078

[B52] Wang G. Z. Zhang W. Fang Z. T. Zhang W. Yang M. J. Yang G. W. (2014). Arsenic trioxide: marked suppression of tumor metastasis potential by inhibiting the transcription factor Twist *in vivo* and *in vitro* . J. Cancer Res. Clin. Oncol. 140 (7), 1125–1136. 10.1007/s00432-014-1659-6 24756364 PMC11823520

[B53] Wang L. Li K. Lin X. Yao Z. Wang S. Xiong X. (2019). Metformin induces human esophageal carcinoma cell pyroptosis by targeting the miR-497/PELP1 axis. Cancer Lett. 450, 22–31. 10.1016/j.canlet.2019.02.014 30771436

[B54] Waxman S. Anderson K. C. (2001). History of the development of arsenic derivatives in cancer therapy. Oncologist 6 Suppl 2, 3–10. 10.1634/theoncologist.6-suppl_2-3 11331434

[B55] Wilcox C. S. (2010). Effects of tempol and redox-cycling nitroxides in models of oxidative stress. Pharmacol. Ther. 126 (2), 119–145. 10.1016/j.pharmthera.2010.01.003 20153367 PMC2854323

[B56] Xia J. Xu H. Zhang X. Allamargot C. Coleman K. L. Nessler R. (2017). Multiple myeloma tumor cells are selectively killed by pharmacologically-dosed ascorbic acid. EBioMedicine 18, 41–49. 10.1016/j.ebiom.2017.02.011 28229908 PMC5405162

[B57] Yoon J. S. Hwang D. W. Kim E. S. Kim J. S. Kim S. Chung H. J. (2016). Anti-tumoral effect of arsenic compound, sodium metaarsenite (KML001), in non-Hodgkin’s lymphoma: an *in vitro* and *in vivo* study. Invest. New Drugs 34 (1), 1–14. 10.1007/s10637-015-0301-z 26581399

[B58] Zerillo J. A. Schouwenburg M. G. van Bommel A. C. M. Stowell C. Lippa J. Bauer D. (2017). An international collaborative standardizing a comprehensive patient-centered outcomes measurement set for colorectal cancer. JAMA Oncol. 3 (5), 686–694. 10.1001/jamaoncol.2017.0417 28384684

[B59] Zhao X. Feng T. Chen H. Shan H. Zhang Y. Lu Y. (2008). Arsenic trioxide-induced apoptosis in H9c2 cardiomyocytes: implications in cardiotoxicity. Basic Clin. Pharmacol. Toxicol. 102 (5), 419–425. 10.1111/j.1742-7843.2007.00150.x 18346055

[B60] Zhou B. Zhang J. Y. Liu X. S. Chen H. Z. Ai Y. L. Cheng K. (2018). Tom20 senses iron-activated ROS signaling to promote melanoma cell pyroptosis. Cell Res. 28 (12), 1171–1185. 10.1038/s41422-018-0090-y 30287942 PMC6274649

